# Lipidomic Characterization of Oocytes at Single-Cell Level Using Nanoflow Chromatography-Trapped Ion Mobility Spectrometry-Mass Spectrometry

**DOI:** 10.3390/molecules28104202

**Published:** 2023-05-19

**Authors:** Pujia Zhu, Guowei Bu, Ruifeng Hu, Xianqin Ruan, Rongrong Fu, Zhourui Zhang, Qiongqiong Wan, Xin Liu, Yiliang Miao, Suming Chen

**Affiliations:** 1 The Institute for Advanced Studies, Wuhan University, Wuhan 430072, China; zhupujia@163.com (P.Z.);; 2 Institute of Stem Cell and Regenerative Biology, College of Animal Science and Veterinary Medicine, Huazhong Agricultural University, Wuhan 430070, China

**Keywords:** mass spectrometry, lipidomic, single-cell, oocytes, liquid chromatography

## Abstract

Mass spectrometry (MS)-based lipidomic has become a powerful tool for studying lipids in biological systems. However, lipidome analysis at the single-cell level remains a challenge. Here, we report a highly sensitive lipidomic workflow based on nanoflow liquid chromatography and trapped ion mobility spectrometry (TIMS)-MS. This approach enables the high-coverage identification of lipidome landscape at the single-oocyte level. By using the proposed method, comprehensive lipid changes in porcine oocytes during their maturation were revealed. The results provide valuable insights into the structural changes of lipid molecules during porcine oocyte maturation, highlighting the significance of sphingolipids and glycerophospholipids. This study offers a new approach to the single-cell lipidomic.

## 1. Introduction

Lipids are major components of cell membranes and intracellular organelles and possess complex biophysical, energy storage, and signaling functions [1,2,3]. Mass spectrometry (MS)-based lipidomic provides a powerful tool for studying cellular metabolism by quantifying the changes of individual lipid classes, subclasses, and molecular species that reflect metabolic differences [1,3,4,5,6,7]. The conventional lipidomic analysis could provide insight into the average lipid composition of cells using an ensemble or complex tissues [4,8]. However, the unique characteristics of individual cells are lost in this process, and the ability to link the individual metabolic characteristics of cells to their respective biochemical functions is impaired [9]. In single-cell analysis, cellular heterogeneity can be revealed, and the biological variability can directly be attributed to individual cells [10,11]. Nevertheless, comprehensive characterization and quantitation of a relatively large number of lipids with complex structures are still difficult at the single-cell level [8,11].

Constrained by the ultra-low quantity of lipids in single cells, most of the current single-cell lipidome analyses are based on direct MS methods. For example, desorption electrospray ionization (DESI) [11,12,13], nano-electrospray ionization (nESI) [9,14], matrix-assisted laser desorption/ionization (MALDI) [15,16,17], and secondary-ion mass spectrometry (SIMS) [18]. However, the lipidome coverage of these methods is far from satisfactory. Liquid chromatography (LC) separation can effectively reduce the ion suppression effect of complex systems and greatly improve the coverage of lipidomic analysis [19]. It also allows the separation of complex isomers in lipids, thus improving the structural resolution of lipidomes [4]. Therefore, liquid chromatography–mass spectrometry remains the promising method for single-cell lipidome analysis [20]. However, regular LC separation uses relatively high flow rates above microliter/min, which is less sensitive for the analysis of very small amounts of samples [21,22]. Meanwhile, the mass spectrometer with both high-speed MS/MS acquisition rate and high sensitivity is also key to achieving high-coverage lipidomic for ultra-trace samples, especially at the single-cell level [22].

As a single cell, the oocyte is the basis of mammalian reproduction [23]. Lipid metabolic reprogramming and its regulation during oocyte maturation are hardwired into the complex developmental program [24,25,26]. However, limitations in obtaining adequate sample amounts and the availability of reliable methods have hindered insights into metabolic patterns and their regulatory mechanisms during oocyte maturation [16]. In addition, the lipid metabolic heterogeneity and unique characteristics of individual oocytes remain to be elucidated [13]. Therefore, a reliable lipidomic method at the single-oocyte level is quite necessary.

**Figure 1:**
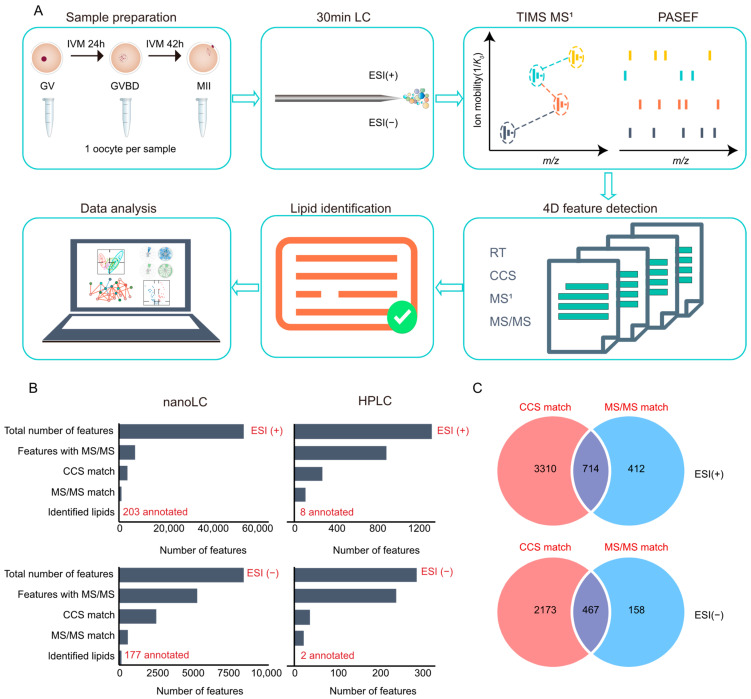
**Overview of the lipidomic at single-cell level.** (**A**) Schematic overview of the workflow for lipidomic profiling in oocytes. (**B**) Sequential data analysis steps used to identify unique lipids from the total number of detected 4D features in single-cell level oocytes. The analysis was performed in both ionization modes using either a nanoLC or an HPLC system. (**C**) Candidate features identified via matching using CCS versus matching using only MS/MS when using the nanoLC system.

Here, a workflow for the high-coverage lipidomic analysis at the single-cell level was developed by combining nanoflow liquid chromatography and trapped ion mobility spectrometry (TIMS)-MS for characterizing lipid metabolism during oocyte maturation (Figure 1A). TIMS-MS is a relatively new mass spectrometric instrument with an MS scan mode termed parallel accumulation serial fragmentation (PASEF), which allows TIMS to be synchronized with MS/MS precursor selection [22]. The coverage of proteomics [27,28] and lipidomic [22,29] can be greatly enhanced due to the increased scan rate of MS/MS without sacrificing sensitivity. Therefore, we reason that this unique benefit of TIMS-MS would facilitate the single-cell lipidomic analysis. Moreover, ion mobility spectrometry could provide additional dimensions for the separation and identification of isomeric lipids. By using the established method, the landscape of lipid metabolism of porcine oocytes during their maturation was demonstrated.

## 2. Results and Discussion

### 2.1. Development of the Single-Cell Lipidomic Workflow Based on nanoLC-TIMS-MS

We aimed to develop a workflow that enables untargeted lipidomic analysis of oocytes in a straightforward manner. To extract lipids from a single oocyte, we followed a modified protocol described by Folch et al. [30]. The scaled-down solvent system (30 μL of methanol, 60 μL of chloroform, and 25 μL of water) was used to alleviate the loss of the lipids. Chloroform is better than methyl tert-butyl ether (MTBE) in this case because it is located in the lower layer and could avoid the evaporation of such a small volume of solvent.

The lipid extract was dried to concentrate and re-dissolved in 15 μL of solvent. Then, 2 μL of the solution was loaded onto a C18 column and eluted within 30 min. The TIMS-TOF Pro mass spectrometer features a dual ion mobility spectrometry analyzer that allows the utilization of up to 100% ofincoming ions. In the PASEF mode, TIMS scan time was set as 100 ms, and the ion current accumulated during 100 ms was compressed into ion mobility peaks of 2–3 ms full width at half maximum (FWHM), which should theoretically result in a 50-fold increase of signal-to-noise compared to continuous acquisition. In addition, multiple precursor ions in each TIMS ramp are selected for fragmentation in data-dependent acquisition (DDA) mode by rapidly switching the quadrupole. These features may enhance the lipidome coverage in lipidomic analysis. For the data processing, the converted data files were imported into MS-DIAL, and the lipids were reliably identified based on the corresponding features extracted from the four-dimensional space (retention time, *m*/*z*, ion mobility, and fragment ion intensity).

To validate the proposed workflow and characterize the lipidome of the oocyte, a pooled lipid solution consists of nine extracts from a single porcine oocyte at different maturation stages (germinal vesicle (GV), *n* = 3; germinal vesicle breakdown (GVBD), *n* = 3; metaphase II (MII), *n* = 3) was prepared and analyzed. Quality control (QC) samples were obtained by mixing single-cell lipid extracts from three periods. As shown in Figure 1C, 714 annotated lipids were identified after matching both MS/MS and CCS in positive ion mode, and 467 of them could be identified in negative ion mode. After further reliability screening, 203 and 177 lipids with high reliability were retained in positive and negative ion modes, respectively. Finally, after the removal of duplicated lipids, a total of 293 lipid molecules with well-defined structures were identified at the single-cell level in the oocytes of both modes (Table S1). In five replicate injections of nano-LC for QC samples, the median relative standard deviation (RSD) was 0.18, and 83.3% of all quantified lipids had an RSD below 0.4. (Figure S1). The identified lipidome shows high coverage and covers major lipid categories, such as glycerophospholipids (phosphatidylcholine (PC), phosphatidylethanolamine (PE), phosphatidylserine (PS), phosphatidylinositol (PI), phosphatidylglycerol (PG), ether PC, ether PE, and ether PI), diacylglycerols (DG), triacylglycerols (TG), ceramides (Cer), and sphingomyelins (SM). In contrast, if HPLC was used instead of nano-LC analysis at the same injection volume and liquid chromatography gradient, only nine lipids were eventually identified (Table S2), indicating the advantage of nano-flow chromatography separation. The above results show the validity and high performance of the developed lipidomic method at the single-cell level, which provides a highly efficient tool for further lipid metabolic study of porcine oocytes during their maturation.

### 2.2. Single-Cell Lipidomic Analysis of Porcine Oocytes at Different Maturation Stages

In many aspects, such as genome, physiology, and disease progression, pigs have a very high degree of similarity to humans. Therefore, the study of porcine oocyte maturation processes can provide important insights into human reproductive development. Lipids are the essential components of porcine oocytes, but the role of the large lipid content throughout oocyte maturation remains unknown.

**Figure 2:**
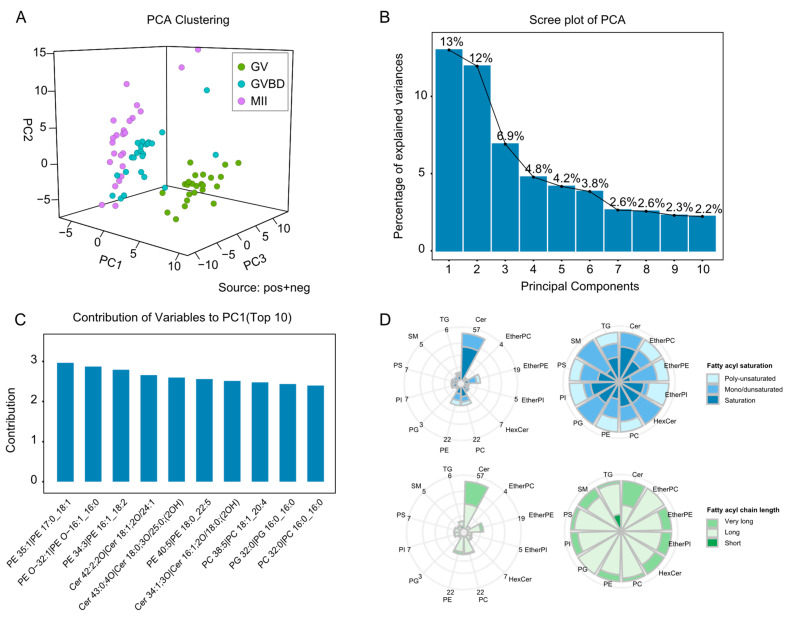
**Single-cell lipidomic analysis of porcine oocytes at different maturation stages.** (**A**) Principal component analysis (PCA) of individual samples. (**B**) Percentage of total variance explained by the top 10 components. (**C**) The top 10 variables contributing to PC-1 are shown separately in the bar plot. (**D**) Radar diagrams illustrate the distribution of fatty acyls of different carbon atom numbers and double bond numbers in major lipid classes for porcine oocyte lipidome. The numbers at the circumferential boundary indicate the number of quantitated lipid species within each lipid class.

In this study, we isolated single porcine oocytes (*n* = 24 per group) at three key time points during meiotic maturation (the arrested GV stage, the GVBD stage after meiotic resumption, and the MII stage at ovulation), and analyzed their lipidome using the developed nanoLC-TIMS-MS technology. QC samples were used to assess the quality of data collected during nano-LC analysis. The injection sequence was random, with one QC sample injected between every 12 samples. We used MS-DIAL software to extract and align ion peaks in positive and negative ion modes and obtained 117,210 and 18,132 ion peaks, respectively. PCA performed on all the samples revealed that the QC samples were clustered in a PCA scores plot (Figure S2), indicating that the stability of the lipidomic analysis was acceptable throughout the run. As shown in Figure 2A–C, PCA analysis using all lipid features shows significant differences among oocytes at different stages. Larger differences were observed between GV and GVBD or MII stages, while smaller differences were observed between GVBD and MII stages. By performing lipidomic analysis of individual oocytes, the heterogeneity exhibited by oocytes at different stages could be observed, and different oocytes from the same developmental stage also showed some differences.

We also successfully identified 182 shared lipid molecules from the oocytes at different stages (*n* = 72), including 96 glycerophospholipids (GPs), 6 glycerolipids (GLs), and 80 sphingolipids (SPs) (Table S3). Retention times of these lipids were reproducible with median CVs of 0.25% in replicate injections prior to alignment (Table S4). The structural characteristics of lipids in porcine oocytes such as chain length and number of double bonds were further analyzed. The results show that the lipids containing long-chain fatty acyls (carbon atom numbers: 16–21) were major components in oocyte lipidome, while short-chain fatty acyls (carbon atom numbers < 16) were mainly distributed in TG. Additionally, sphingolipids such as Cer, SM, and hexosylceramide (HexCer) were primarily composed of saturated or mono-unsaturated very long chain fatty acyls (carbon atom numbers > 21) (Figure 2D).

### 2.3. Lipid Changes during Oocyte Maturation

**Figure 3:**
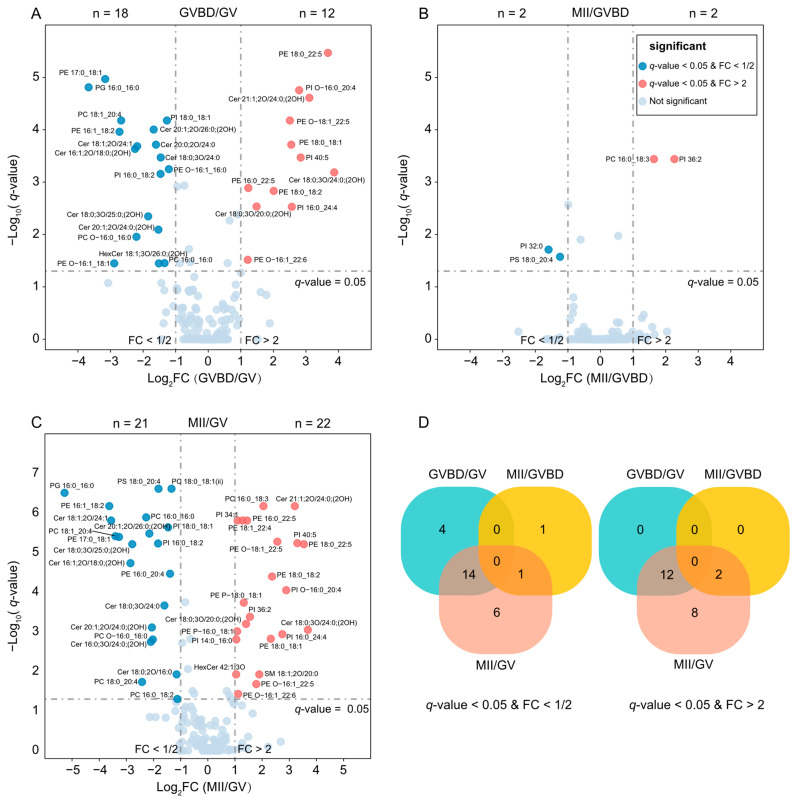
**Differences of the lipids in porcine oocytes with different maturation stages.** (**A**–**C**) Volcano plots display top lipids that were most significantly different in each pairwise comparison between mitochondrial lipidome at GVBD relative to GV, MII relative to GVBD, and MII relative to GV, based on magnitudes of *q*-value and fold changes. Two-sided Nemenyi test was used for post hoc pairwise comparisons, *n* = 24 independent oocytes for each maturation stage. (**D**) The number of lipids that produced significant differences between two comparisons at GV, GVBD, and MII periods.

During the three stages of porcine oocyte maturation, we employed the Kruskal–Wallis test to analyze the differential lipid molecules and used the Nemenyi test to conduct multiple comparisons. To control type I errors, we corrected the *p*-values with the Benjamini–Hochberg method, yielding *q*-values. Based on the results of the data analysis, we identified differential lipid molecules with *q*-values < 0.05 and fold change (FC) ≥ 2 or ≤0.5. We visualized the identified molecules using a volcano plot (Figure 3).

During the GV to GVBD transition, we observed 30 differentially expressed lipid molecules (Table S5), of which 18 were downregulated and 12 were upregulated (Figure 3A). On the other hand, only four differential lipid molecules were detected during the GVBD to metaphase II (MII) transition, with two upregulated and two downregulated (Figure 3B, Table S6). Furthermore, during the GV to MII transition (Figure 3C), we identified a total of 43 differentially expressed lipids (Table S7), 21 of which were downregulated and mainly consisted of ceramides (Cer) and phosphatidylcholines (PC). Specifically, 14 downregulated lipids changed during the GV to GVBD transition, 1 lipid changed during the GVBD to MII transition, and 6 lipids gradually decreased throughout the entire GV to MII process. The remaining 22 lipids were upregulated and mainly consisted of phosphatidylethanolamines (PE), phosphatidylinositols (PI), and ether-linked PE (EtherPE). Among them, 12 lipids were upregulated during the GV to GVBD transition, 2 lipids were upregulated during the GVBD to MII transition, and 8 lipids gradually increased throughout the entire GV to MII process.

Notably, the number of differentially expressed lipid molecules was much higher during the GV to GVBD transition than during the GVBD to MII transition (Figure 3D), indicating that dynamic changes in porcine oocyte lipid metabolism are more significant during the GV to GVBD stage. These findings were consistent with the results of principal component analysis (PCA) clustering and further support our conclusion. The data provide significant insights into the differential lipid metabolism of porcine oocytes during different maturation stages.

### 2.4. Differential Correlation Network Analyses of Lipids during Oocyte Maturation

**Figure 4:**
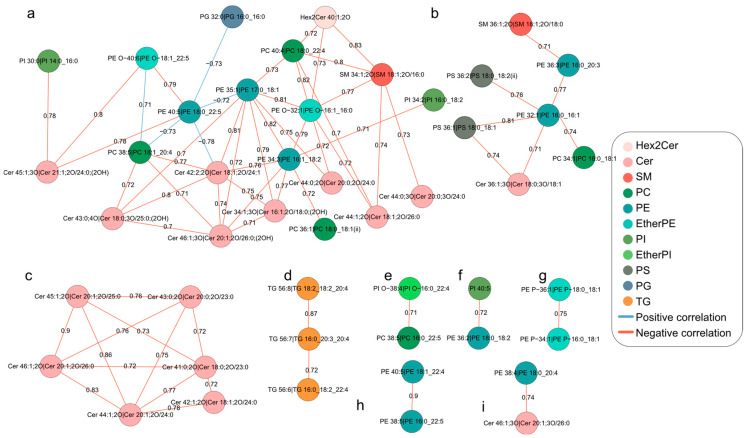
**Differential correlation network analyses of lipids during oocyte maturation.** (**a**–**i**) Nine of the divided independent correlation networks. Lipid pairs with significant differential correlations (*q*-value < 0.05) were connected by lines of different colors according to their changes in correlation patterns.

In order to investigate the potential correlation and metabolic independence between lipid molecules, we screened lipids with Pearson correlation coefficients greater than 0.7 based on the total number of identified lipids to ensure the stability and robustness of the constructed correlation network (Figure 4). The resulting network consisted of a total of 68 edges and 47 nodes, including 16 Cer, 1 Hex2Cer, 2 SM, 5 PC, 10 PE, 3 EtherPE, 3 PI, 1 EtherPI, 2 PS, 1 PG, and 3 TG. Positive and negative correlations between lipids were shown by blue and pink connecting lines, respectively. The network can be divided into 9 independent correlation networks, where network a consisted of most sphingolipids (Cer, Hex2Cer, and SM) and some PE, EtherPE, PC, and PI, network b consisted of most PS and some PE, PC, Cer, and SM, all lipids in network c were Cer, and all lipids in network d were TG. This suggests that they play a role in regular metabolic independence during porcine oocyte maturation. Sphingolipids and glycerophospholipids were intertwined throughout the network, suggesting that they are closely related to the extensive transformation of other lipid classes during porcine oocyte maturation.

## 3. Experimental Section

### 3.1. Chemicals and Materials

The experimental reagents used in this study included 1-butanol (BuOH) and methyl tert-butyl ether (MTBE) were purchased from Innochem (Beijing, China). Ammonium formate, phosphoric acid, hyaluronidase, mineral oil, and protease were purchased from Sigma-Aldrich (St. Louis, MO, USA). Isopropanol (IPA), methanol (MeOH), and acetonitrile (ACN) were purchased from Thermo Fisher Scientific (Waltham, MA, USA). Chloroform was purchased from Yonghua Chemical (Changshu, China), and formic acid was purchased from Aladdin (Shanghai, China). Ultra-pure water was made in-house by Millipore Direct-Q5. All reagents were of analytical grade or higher and were used as received without further purification.

### 3.2. Oocyte Recovery

The experiments were conducted following the guidance of the Animal Care and Use Committee of Huazhong Agriculture University. The ovaries were obtained from a local slaughterhouse in Wuhan, China, stored in 0.9% (*w*/*v*) saline containing penicillin, and transported to the laboratory within 2 h. The follicular fluid was extracted from 2–6 mm diameter follicles using a 10 mL disposable syringe (18G) and collected into a 15 mL centrifuge tube. After allowing cumulus-oocyte complexes to precipitate, the cumulus-oocyte complexes (COCs) were selected under a stereomicroscope and washed with in vitro maturation (IVM) culture medium [31]. The COCs were then transferred into mineral oil-covered 96-well plates containing IVM medium and placed in a 5% CO_2_ incubator at 38.5 °C. Each well contained approximately 30 COCs.

The oocytes were collected at GV (germinal vesicle), GVBD (germinal vesicle breakdown), and MII (metaphase II) stages at 0 h, 24 h, and 42 h of incubation, respectively. Before collection, cumulus cells were removed from cumulus-oocyte complexes (COCs) using hyaluronidase. The zona pellucida of the oocytes were then removed with 0.25% (*w*/*v*) pronase, followed by three washes with PBS. Finally, oocytes were transferred with minimal liquid (<0.5 μL) into low-adsorption RNase-free PCR tubes and stored at −80 °C until lipid extraction. Twenty-four biological replicates of each stage were performed.

### 3.3. Lipid Extraction

To carry out the lipid extraction process at a low temperature, an ice box was prepared. The PCR tube containing one porcine oocyte was first filled with 30 μL of methanol and then vortexed for 30 s. Afterward, the solution in the tube was sonicated in an ice bath for 2 min. Subsequently, 60 μL of chloroform was added into the tube, and the mixture was vortexed for 30 s and sonicated in an ice bath for 4 min. After that, 25 μL of ultra-pure water was added, and the sample was sonicated in ice water for another 5 min. Finally, the mixture was centrifuged for 10 min at 3000 rpm at 4 °C. The lower layer was transferred to a new 0.2 mL centrifuge tube and blown dry under nitrogen. The resulting dried lipid extract was then re-dissolved in 15 μL of mixed solvent (BuOH: IPA: H_2_O = 8:23:69 (*v*/*v*/*v*) with the addition of 5 mM phosphoric acid) and stored at −20 °C until assayed.

### 3.4. Liquid Chromatography

The chromatographic separation was performed using a nanoflow chromatography system (EASY-nLC™ 1200, Thermo Fisher Scientific, New York, NY, USA). The analytical column was prepared in the laboratory by packing C18 resins (1.9 μm, Dr. Maisch, Ammerbuch-Entringen, Germany) in a pulled tip silica capillary tube (75 μm I.D., 20 cm length), and the column length was adjusted to 15 cm. The mobile phase A was prepared by mixing acetonitrile and water in a ratio of 6:4 (*v*/*v*), with 10 mM ammonium formate and 0.1% formic acid added as an additive solvent mixture. The mobile phase B was prepared by mixing isopropanol and acetonitrile in a ratio of 9:1 (*v*/*v*), with 10 mM ammonium formate and 0.1% formic acid added as an additive solvent mixture. For lipid separation, a linear gradient was run at a flow rate of 300 nL/min. The gradient started at 0% B and raised to 30% B at 3.0 min, then to 51% B at 7.0 min, to 61% B at 12.0 min, to 71% B at 17.0 min, to 99% B at 22.0 min, and held until 27.0 min, followed by a decrease to 1% B at 28.0 min until 30.0 min. The column chamber was set at 60 °C, and the injection tray temperature was set at 7 °C. A 2 μL injection volume was used.

### 3.5. Trapped Ion Mobility Spectrometry-Mass Spectrometric Analysis

We utilized a TIMS-TOF Pro mass spectrometer (Bruker, Bremen, Germany) equipped with a PASEF acquisition mode to perform MS detection in both positive and negative ion modes. Mass spectra were acquired within the range of *m*/*z* 50–1550 and ion mobility range of 0.6 to 1.95 Vs/cm^2^. The average mass resolution is 67,000 at *m*/*z* 1222. Each scan cycle consists of one full TIMS-MS scan and three PASEF MS/MS scans with a total duration of 0.42 s. Precursor ions with intensity above a threshold of 100 but below 4000 were repeatedly scheduled, and those not meeting the criteria were dynamically excluded for 0.2 min. The collision energy was linearly increased from 25 to 45 eV in positive mode and from 35 to 55 eV in negative mode.

### 3.6. Data Analysis and Bioinformatics

To perform a quantitative and qualitative analysis of lipids, we initially viewed the total ion current (TIC) map of the sample using DataAnalysis software (Bruker). The data files generated in .d format were converted to .ibf format using the IbfConverter file converter and then imported into MS-DIAL (version 4.9.1) for data processing, including peak extraction, alignment, and annotation. To ensure accuracy, only peaks appearing in at least 80% of mixed samples were considered for further analysis. Identification of lipids was achieved by searching the internal theoretical MS/MS spectra library of MS-DIAL, with the accurate mass tolerances set as 0.01 Da for MS^1^ and 0.05 Da for MS^2^. The resulting matrices contain lipid names and peak area information for all samples in both positive and negative ion modes. The matched lipids were manually identified based on the characteristic ions [5].

The *m*/*z* values, retention times, and CCS values of the MS^1^ mass spectrum were used to align the mass spectra of single-cell samples, with a tolerance of ±0.01 Da, 2 min, and 10 Å^2^, respectively. Relative quantification was then performed using qualitative results obtained through alignment and matching. Principal component analysis (PCA), non-parametric tests, multiple comparisons, and correlation analysis were conducted using R-4.2.0.

### 3.7. Lipid Identification and Report

Firstly, the IbfConverter file converter was used to convert the raw data files (.d) obtained from positive and negative ion modes into the ibf format. Subsequently, the ibf files were imported into MS-DIAL (version 4.9.1) for data processing, including peak extraction, alignment, and annotation to obtain a list of candidate lipids. This software provides a comprehensive overview of the lipidome, including retention time (RT), collision cross-section (CCS), lipid subclasses, as well as ion mobility, MS/MS spectra, and other MS information.

In the candidate list, more candidate features can be identified if matching is performed using only CCS compared to matching using only MS/MS. To ensure the reliability of the identification, we retained those features that could match CCS, *m*/*z*, and MS/MS spectra in all replicate injections among the thousands of features detected. Subsequently, we manually evaluated all automatically annotated features’ MS/MS spectra and checked whether they conform to the ECN model to confirm the correctness of lipid identification [5,32].

## 4. Conclusions

In this study, we accomplished large-scale identification and relative quantification of oocyte lipids at the single-cell level by utilizing nano-flow LC in combination with TIMS-MS with PASEF mode, which provides a highly efficient tool for further lipid metabolic study of oocytes. Using this approach, lipid changes in porcine oocytes during maturation were demonstrated. The distinct lipidome landscapes show the comprehensive changes in bioactive lipids of oocytes at the GV, GVBD, and MII stages. These molecular signatures provided deep insight into oocyte maturation.

## Data Availability

Data on identified lipids can be found in Supplementary Tables. Other data supporting this study are available from the corresponding author upon reasonable request.

## Supplementary Materials

The following supporting information can be downloaded at: https://www.mdpi.com/article/10.3390/molecules28104202/s1, Figure S1: RSD of 293 lipids quantified in five replicate injections of nanoLC for QC samples; Figure S2: Total PCA scores of all samples; Table S1: Lipids identified with nanoLC-MS systems; Table S2: Lipids identified with HPLC-MS systems; Table S3. Identification of lipidomic lipids in porcine oocytes; Table S4: CV of retention times of the identified lipids; Table S5: Volcanic plot data-GVBD vs. GV; Table S6: Volcanic plot data-MII vs. GVBD; Table S7: Volcanic plot data-MII vs. GV.
