# Supplementary material for: Lipidomic Characterization of Oocytes at Single-Cell Level Using Nanoflow Chromatography-Trapped Ion Mobility Spectrometry-Mass Spectrometry
Source: Molecules. 2023 May 19;28(10):4202. doi: 10.3390/molecules28104202 (PMC10221703; doi:10.3390/molecules28104202)
Supplement: Supplementary file 1 [file molecules-28-04202-s001.zip › Supplementary Information.pdf]

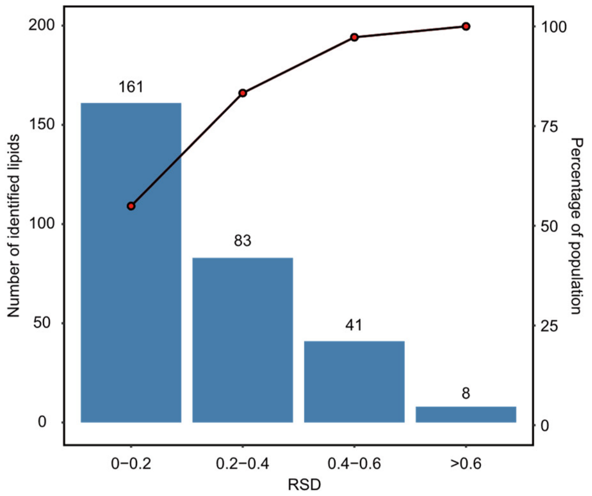

Figure S1. RSD of 293 lipids quantified in five replicate injections of nano-LC for QC samples.

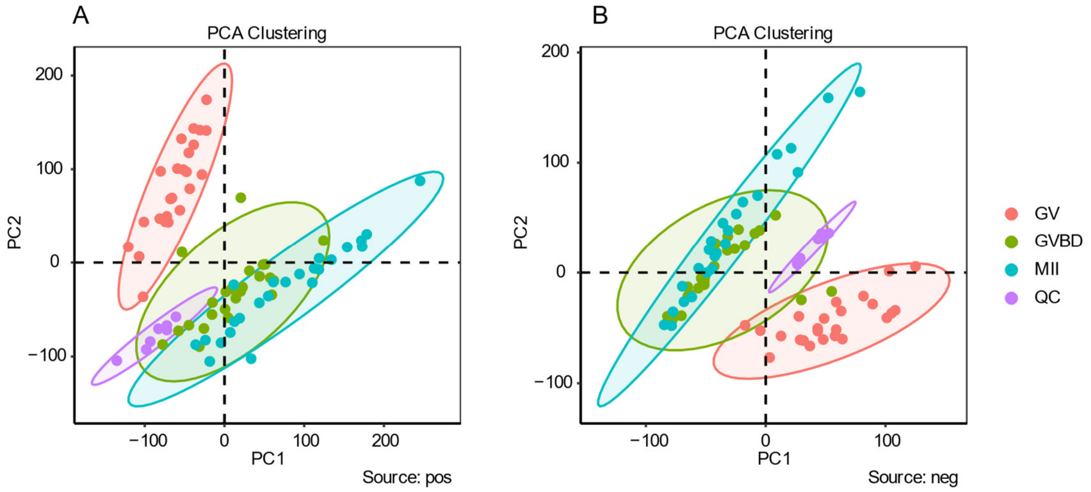

Figure S2. Total PCA scores of all samples. (**A**) QC and PCA in positive ion mode; (**B**) QC and PCA of experimental samples in negative ion mode.
